# Methotrexate, an anti-inflammatory drug, inhibits Hepatitis E viral replication

**DOI:** 10.1080/14756366.2023.2280500

**Published:** 2023-11-17

**Authors:** Akash Kumar, Preeti Hooda, Anindita Puri, Radhika Khatter, Mohammed S. Al-Dosari, Neha Sinha, Mohammad K. Parvez, Deepak Sehgal

**Affiliations:** a Department of Life Sciences, Virology lab, Shiv Nadar Institution of Eminence, Greater Noida, India; b Department of Pharmacognosy, College of Pharmacy, King Saud University, Riyadh, Saudi Arabia; c Department of Infectious Diseases and Microbiology, School of Public Health, University of Pittsburgh, Pittsburgh, PA, USA

**Keywords:** Hepatitis E virus, helicase, RNA replication, antiviral, methotrexate

## Abstract

Hepatitis E Virus (HEV) is a positively oriented RNA virus having a 7.2 kb genome. HEV consists of three open reading frames (ORF1-3). Of these, ORF1 codes for the enzymes Methyltransferase (Mtase), Papain-like cysteine protease (PCP), RNA helicase, and RNA-dependent RNA polymerase (RdRp). Unavailability of a vaccine or effective drug against HEV and considering the side effects associated with the off-label use of ribavirin (RBV) and pegylated interferons, an alternative approach is required by the modulation of specific enzymes to prevent the infection. HEV helicase is involved in unwinding the double-stranded RNA, RNA processing, transcriptional regulation, and pre-mRNA processing. Therefore, we screened FDA-approved compounds from the ZINC15 database against the modelled 3D structure of HEV helicase and found that methotrexate and compound A (Pubchem ID BTB07890) inhibit the NTPase and dsRNA unwinding activity leading to inhibition of HEV RNA replication. This may be further authenticated by *in vivo* study.

## Introduction

Hepatitis E Virus (HEV), the primary causative agent of acute hepatitis, is a quasi-enveloped virus and belongs to the family Hepeviridae. Hepatitis E leads to ∼20 million infections with an overall 3.3% death rate, including 20–30% mortality in pregnant women annually^1^. It has a positive-stranded RNA genome of ∼7.2 kb, which codes for three major open reading frames (ORF1-3). An additional ORF, i.e. ORF4, has been observed in genotype 1, which enhances the viral replication under the endoplasmic reticulum (ER) stress. While ORF1 encodes the non-structural protein essential for viral replication and invasion of the host immune system, ORF2 encodes the viral capsid protein, and ORF3 encodes a small phosphoprotein essential for virion egress. ORF1 is the largest, which encodes seven domains, namely: Methyltransferase (MTase), Y-domain, X-domain, Papain-like cysteine protease (PCP), Hypervariable region (HVR), RNA Helicase (Hel/NTPase) and RNA-dependent RNA polymerase (RdRp)^2^. However, whether the ORF1 acts as a single polyprotein or gets processed into functionally active components is still debated. Since helicase participates in myriad molecular events associated with DNA and RNA metabolism, it is an essential drug target. Many RNA viruses presumably encode their helicases to remove partial duplexes within the genome and facilitate viral replication directly or indirectly^3^. Therefore, the functional importance of helicases means that their inhibitors or modulators are potentially important as therapeutic agents. Given this, viral helicases are among the most critical drug targets for developing effective antivirals.

Because of self-limiting acute manifestation in the general population, there has been no established treatment for hepatitis E. However, with the recent emergence of chronic infections, interferon-*α* (pegIFN-*α*-2a) and ribavirin (RBV) have become the off-label drugs of choice^4^^,^^5^but the chances of treatment failure are also high. In this study, therefore, potential small molecule inhibitors of HEV helicase were virtually identified using homology modelling, molecular docking, and MD simulation tools and validated using *in vitro* and HEV transient culture.

## Material and methods

### Modelling of HEV helicase

With a 24.90% identity with tomato mosaic virus helicase (PDB ID:3VKW)^6^, the 3D structure of HEV helicase as its closest homolog was modelled using (Iterative ASSembly Refinement server (I-TASSER)^7–9^. Alpha Fold^10^ was also used to predict the 3D structure of HEV helicase, showing TMV helicase as its closest homolog. Based on the Z-score, the best predicted 3D model was chosen and improved using the protein preparation wizard of Maestro (Schrodinger, Schrodinger suite, LLC, New York, NY), following molecular dynamics (MD) simulation at 200 ns, utilising the OPLS3a force field^11^. The protein domain prediction study was carried out using the InterPro web service^12^. The protein’s binding site was predicted by using the COACH web server^13^. Based on the two techniques (TM-SITE and S-SITE), the server received a predicted helicase 3D structure as an input file. These techniques use the BioLip protein function database^14^ to find templates for binding ligands^14^. In addition, COFACTOR^15^ and 3D Ligand Site^16^ were employed to predict the ligand binding site in the modelled helicase.

### Virtual screening for FDA-approved compounds

The 3D structures of 136 FDA-approved compounds were retrieved from the ZINC database (https://zinc12.docking.org/browse/catalogs/natural-products). Energy minimisation was applied to all the ligands once imported into OpenBabel^17^using the PyRx program^18^^,^^19^. Using the Universal Force Field (UFF)^20^by conjugated gradient algorithm, the energy minimisation (EM) of the imported chemicals was performed. The number of steps used for the update was set to 1, and a total of 200 steps were set for EM. Moreover, the EM of the ligands was programmed to terminate at a difference in energy of 0.1 kcal/mol. The energy-minimised structures were transformed into the Autodock PDBQT format for molecular docking. Using the YASARA EM server, the predicted protein 3D structure’s energy was minimised^21^. Also, the protein was prepared using maestro Schrodinger. Briefly, the protein production wizard’s Epik interface created the tautomeric and ionisation states of the residues and added hydrogens. The protonation states of histidine were discovered through their optimisation using a hydrogen bonding network.

Autodock vina^22^ (PyRx version 0.8, San Diego, CA 92101) was used to execute molecular docking. The search space was configured as centre (x,y,z) = (−40.4675382157, −21.7091574583, and −33.4514330364) and size (x,y,z) = (27.828740215 and 26.484244255). The top hit with the lowest binding energy was selected with an exhaustiveness of 8 in the molecular docking simulation. The Biovia Discovery studio visualiser (BIOVIA, Dassault Systèmes, San Diego, CA, 2021) was used to create 2D interaction diagrams of the docked ligands. The lowest binding energy, number of hydrogen bonds, 2D interaction diagram of the protein-ligand complex, and literature reference were used as search criteria, filtering to five compounds: Methotrexate, 5-methyltetrahydrofolate (levomefolic acid), daunorubicin HCL, prednisolone, and disodium cromoglycate.

### MD simulation

Desmond^23^ (Schrödinger Release 2023–2: Desmond Molecular Dynamics System, D. E. Shaw Research, New York, NY, 2021) was used to conduct MD simulations for HEV helicase refined structures in Apo and complexed with specific ligands for 200 ns. A preset SPC solvent model was selected for system builder^24^. The orthorhombic boundary in which the apo and protein-ligand complexes were constructed had dimensions of 10 × 10 × 10. By adding sodium ions, the system’s negative charge was balanced, and the MD simulation was run for 200 ns at a constant NPT ensemble temperature of 300.0 K and a constant pressure of 1 bar. To determine the root-mean-square deviation (RMSD) and root-mean-square fluctuations (RMSF), the simulation trajectories were recorded at 100 ps and 1000 frames.

### HEV helicase cloning, expression, and purification

The HEV helicase (735 bp) was amplified from the HEV (genotype 1; accession no. AF444002.1) cDNA clone pSK-HEV-2^23^^,^^25^ using the forward and reverse primers sets (5′agcc|atatgggttgccgtgtgacc3′ and 5′gtgc|tcgagttaaaagaagttgttcac3′). Following standard laboratory procedures, the amplified gene was cloned in the pET28a vector between *NdeI* and *Xho I* restriction sites. The cloning was verified using colony PCR using Nde *I* and *Xho I* (New England BioLabs, Ipswich, MA) double digestion and DNA sequencing. The recombinant plasmid was further transformed to BL21(DE3) using the routine protocol for expressing HEV helicase. The transformed recombinant BL21 cells were expanded until the culture’s optical density (OD_600 nm_) reached 0.6 before being induced with 0.4 mM IPTG (Sigma Aldrich, St. Louis, MO) at 26 °C for 4 h. The protein solubilisation was carried out as described previously^26^. The Immobilised Metal Affinity Chromatography (IMAC) was used to purify the solubilised protein, and the protein was eluted in a buffer containing 10 mM Tris–Cl (pH 8.0), (Sigma Aldrich), 100 mM NaCl (Sigma Aldrich), 250 mM imidazole, and 0.1% N-lauryl Sarcosine (NLS; Sigma Aldrich). Further, the buffer of purified protein was exchanged with 10 mM Tris–Cl (pH 8.0), 100 mM NaCl, and 0.08% NLS before proceeding with enzyme assays.

### NTPase activity assay

The NTPase activity of the HEV helicase enzyme was performed using a colorimetric-based Malachite green test^27^. The assay followed the manufacturer’s instructions The malachite green assay was performed as per the manufacturer’s protocol (Sigma Aldrich) cat no MAK307-1KT. The assay operates on the phosphate release from the reaction between malachite green and molybdate (AM/MG reagent) as the basis for complexation^27–30^. Briefly, an 80 µL reaction mixture containing 100 pmol of pure helicase protein, 0.4 mM GTP (Sigma Aldrich), 10 mM Tris (pH 8.0), and 100 mM NaCl, and Milli-Q water was incubated at room temperature (RT) for 10 min, and 20 µL of working reagent was added to the reaction mixture. The absorbance was measured on plate reader and a standard curve was generated using known concentrations of phosphate ranging from 0 to 44 uM to correlate absorbance with released phosphate concentration. The absorbance was plotted on *Y*-axis against the phosphate concentration on *X*-axis, and a straight-line equation (*Y* = 0.02182 * *X* + 0.04158) was determined using linear regression in GraphPad Prism version 9.0 (Boston, MA). The graphs were plotted as a function of the concentration of phosphate released. The effects of pH, temperature, enzyme concentration, and substrate concentration on helicase enzyme activity were studied to optimise enzyme activity^31^.

### Double-stranded RNA unwinding assay

A fluorescently tagged RNA HRNA1 5′-CCAGGCGACAUCAGCG-3 and HRNA2 5′-[6FAM] UUUUUUUUUUUUUUCGCUGAUGUCGCCUGG-3 were commercially synthesised (Symbio Technologies, Monmouth Junction, NJ) to determine the unwinding of HEV double-stranded RNA^32^. The unwinding activity of the purified HEV helicase was evaluated under various conditions, including varying enzyme and substrate concentrations. The reaction mixture for the unwinding assay consisted of strand displacement buffer (50 mM HEPES, pH 7, 2 mM MgCl_2_, 0.05 mg BSA/mL, 2 mM DTT, and 0.4 mM ATP)^32^, 200 nM dsRNA, and 0.89 µM of purified helicase was incubated for 1 h at RT and, the fluorescence was measured on Tecan Spark^®^ (Tecan Trading AG, Männedorf, Switzerland) multimode plate reader with filter for 5′-[6FAM]. GraphPad Prism software (Boston, MA) was used to plot the graphs, and all the reactions were performed in triplicates. To confirm the unwinding activity of the helicase, 200 nM dsRNA was incubated with 0.4 mM ATP, 0.2 µM MgCl_2_, and increasing concentration of Helicase ranging from 500 nM to 5 µM.

### Enzyme inhibition assay

Based on their docking score, hydrogen bonds, and protein–ligand interaction, the five selected compounds from virtual screening were tested for their ability to suppress NTPase activity. The putative inhibitors’ concentrations ranged from 0 to 10 µM during the optimisation-focused inhibition tests on the purified protein’s NTPase activity. In a nutshell, GTP was added after inhibitors were incubated with 100 pmol of the purified helicase at RT for 10 min. The working reagent was added after the reaction mixture had been incubated at RT for 10 min, and the absorbance was measured at 600 nm.

Similarly, the inhibition of pure helicase’s unwinding activity was investigated in the presence of other inhibitors at concentrations ranging from 0 to 10 µM. The reaction was set up as described above, and the fluorescence was measured at 485 excitation wavelength and 535 nm emission wavelength. The fluorescence was measured using Tecan Spark^®^ (Tecan Trading AG, Switzerland) multimode microplate reader software using. Helicase unwinding activity inhibition by methotrexate was also confirmed using Native PAGE. An increasing concentration of methotrexate was added to the unwinding assay reaction described above and kept at RT for 2.5 h. All the samples were mixed with 6X non-denaturing dye and loaded onto a 10% NATIVE PAGE.

### Microscale thermophoresis

Microscale Thermophoresis (MST) was performed to study the binding affinity of selected compounds with HEV helicase, as mentioned previously^33–36^. Briefly, the purified enzyme was fluorescently labelled using a lysine labelling kit (RED-NHS 2nd Generation; Nanotemper Technologies, San Francisco, CA). Sixteen samples of ligands were prepared in 2-fold serial dilution ranging from 10 µM to 150 pm while the concentration of helicase was kept constant at 50 nM. The ligand was mixed with fluorescently labelled protein at a 1:1 ratio. Subsequently, the samples were Loaded in 16 glass capillaries in the capillary tray and analysed using Monolith NT.115 analysis software (Nanotemper Technologies).

### Cell culture

Huh 7 cells were maintained in DMEM medium (Dulbecco’s modified eagle medium, Gibco, Grand Island, NY) supplemented with 10% Foetal bovine serum (FBS, Gibco) and 1x Pen-Strep (Thermo Fisher, Waltham, MA) in a 5% CO_2_ humidified incubator at 37 °C.

### Toxicity assay

To study the cell viability, Huh7 cells were grown in Dulbecco’s Modified Eagle Medium (DMEM, Gibco^™^, Life Technologies, Grand Island, NY), supplemented with 10% Foetal Bovine Serum (FBS, Gibco^™^, Qualified, Brazil origin). Further, Huh7 cells were seeded in a 96-well plate (∼0.7 × 10^4^ cells/well) and incubated at 37 °C in a humidified 5% CO_2_ incubator. After 24 h, the media was replaced, having varied concentrations of methotrexate, the identified drug with maximum potency, ranging from 0 to 500 μM. After 4 d, the MTT (3-dimethylthiazol-2-yl-diphenyl tetrazolium bromide) assay was performed per the manufacturer’s protocol (Himedia Laboratories, Thane, India). Cell viability percentage was calculated by comparing treated cells with untreated cells.

### In vitro transcription and transfection

For transfection, pSHEV-3 (cDNA clone of HEV genotype 3; accession no. AY575859.1) was linearised using *Xba I* (New England BioLabs). The helicase region of GT1 and GT3 share greater than 90% sequence homology Sub-genomic HEV carrying luciferase gene (accession number JQ679013) was linearised for replicon assay with *MluI*. The linearised DNA was subjected to *in vitro* transcription, as mentioned previously^26^^,^^36^, and the RNA was transfected in Huh7 cells using Lipofectamine 3000 (Invitrogen, Carlsbad, CA). Briefly, a day before transfection, approximately 0.8 × 10^6^ cells were seeded in each well of a six-well plate, and for HEV replicon assay, ∼5–7 × 10^3^ cells were seeded in each well of 96 plate. After 24 h, the media was removed, the cells were washed twice with Opti-MEM media (Gibco^™^, Grand Island, NY), and the transfection mixture was prepared as mentioned earlier^25^^,^^26^. The cells were incubated with a transfection mixture for 6–8 h at 37 °C. The transfection mixture was then replaced by DMEM complete medium with different concentrations of methotrexate and compound A.

### HEV replicon assay

Following transfection, the cells were incubated with inhibitors for 72 h at 37 °C in a CO_2_ incubator. Renilla Luciferase Assay kit (Promega, Madison, WI) was used to perform the luciferase assay. The assay was performed as per the manufacturer’s protocol. Briefly, the media was discarded, and the cells were washed twice with PBS to ensure the removal of any unbound RNA. The cells were then lysed according to the manufacturer’s protocol, and the luminescence was recorded on Tecan Spark^®^ (Tecan Trading AG, Switzerland) multimode plate reader. In replicon, the luminescence was normalised w.r.t to uninfected (0%) and untreated cells (100%).

### Quantification of transfected RNA

The transfected cells were harvested 4 d post-transfection and washed twice with 1x PBS to remove any unbound viral RNA. As mentioned, the cellular RNA from uninfected, untreated, and treated cells was isolated using TRIzol Reagent. Briefly, the isolated RNA was subjected to DNase I treatment. The RNA was purified using the column purification method (RNeasy MinEluteCleanup Kit, Qiagen, Hilden, Germany), and cDNA synthesis was performed per the manufacturer’s protocol (Verso cDNA synthesis Kit, Invitrogen). Viral RNA was quantified using the standard equation, as mentioned previously^36^.

### Immunofluorescence assay (IFA)

For IFA, the cells were grown on a 96-well plate and transfected using Lipofectamine, as mentioned above. Post 6-8h transfection, the mixture was replaced with complete DMEM medium in the untreated well, and 100 nM of methotrexate was added to another well. The cells were incubated for 4–5 d, and subsequently, the media was removed, and the cells were carefully washed twice with 1X PBS. The cells were fixed with 4% paraformaldehyde (PFA) and incubated for 5–10 min at RT. After fixing, the cells were again washed with 1× PBS, and the permeabilisation was performed using PBS with 0.1% Triton-X and 0.1% BSA (Bovine Serum Albumin, HiMedia, Mumbai, India). The blocking was performed using 3% BSA for 1 h at RT. After blocking, the cells were incubated with anti-HEV-ORF2 (Dilution, 1:400) antibody against the HEV capsid protein for 1 h at RT. The cells were washed thrice with PBS-T (Tween-20, 0.05%, Sigma Aldrich). The Goat-Anti-Rabbit Alexa Fluor 488 (Thermo Fisher) was used as a secondary antibody, and the cells were incubated for 30 min in the dark. The cells were washed thrice with PBS-T and stained with DAPI (Invitrogen^™^). The images were acquired on ImageXpress Micro Confocal High-Content Imaging System (Molecular Devices, San Jose, CA) using FITC and DAPI channels with a 10 X objective lens. Sixteen fields per well were acquired to cover the whole well area. The multi-wavelength cell scoring module of the MetaXpress Software was used for the analysis.

## Results

### In silico analysis of HEV helicase

**Figure 1.**
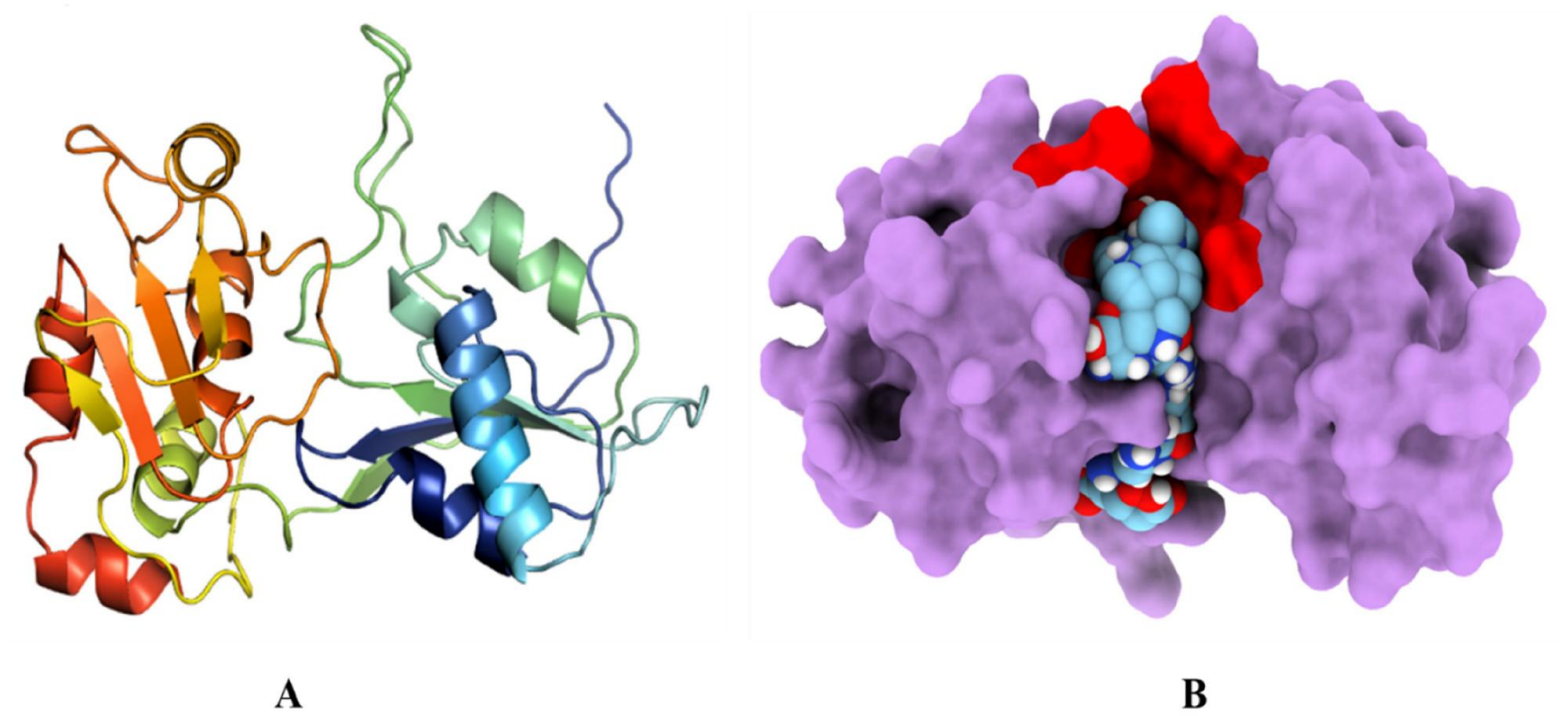
*In silico* analysis of HEV helicase. (A) Represents the ribbon structure representation of The HEV helicase modelled structure. (B) Represents the P-loop containing the Helicase domain (85–232 amino acids residues) coloured in red (as predicted by the InterPro web server); the ball-shaped structure represents the GTP-bound binding pocket of helicase. The binding pocket residues are highlighted in red.

TMV helicase homology modelling predicted the 3D structure of HEV helicase using I-TASSER (Figure 1(A)). Pairwise sequence alignment between the TMV and HEV Helicase identified several conserved key motifs of superfamily-1 helicases^37^. A list of such motifs, along with their function and locations, is given in Table 1 below. Motif I (GXPGTGKT) Residues are involved in NTP binding. They are present between β1 and the alpha helix domain, forming a loop, with only a single amino acid substitution (S–C) in HEV Helicase compared to TMV Helicase. Similarly, in motif II (YDEXXQ), which is also a part of the NTP binding site, residues such as IDE were present in HEV helicase. Other conserved motifs identified by sequence alignment between helicases of TMV and HEV are given in Table 1. Domain analysis of HEV helicase using the InterPro web server also showed the presence of a P-loop containing nucleoside triphosphate hydrolases (amino acid no. 85–232) (Figure 1(B)). Further, the COACH web server was used to identify potential binding site residues, and the results were sorted based on the C-score. The most probable binding site of HEV helicase with a C-score of 0.53 and cluster size of 26 is formed by Pro18, Gly19, Ser20, Gly21, Lys22, Ser23, Arg24, Gln98, His127, Arg128, Gly186, Thr188, and Arg217. The additional information regarding the binding site is given in Supplementary data (Table S1 and Figure S1). The prediction of possible critical site residues also showed the role of conserved motifs in forming a binding cleft. Amino acid residues GVPGSGKSR are a part of motif I and play an essential role in NTP binding in SF-1 helicases^6^^,^^37^.

**Table 1.** Conserved motifs of SF1 helicases in TMV and HEV Helicase.

Motif	Consensus	TMV helicase	HEV helicase	Function
I	GxPGTGKT	GVPGCGKT	GVPGSGKS	NTP binding site
Ia	xxTNxAx	VPGRQAA	VPTRELR	Nucleic acid binding site
to	YDExxQ	IDEGLM	IDEAPSL	NTP binding site
III	GDxxQLxx	GDTQQIPYINR	GDPNQIPAIDF	Coordination between NTP and nucleic acid binding site
IIIa	xxYRx	TTLRC	VTHRCPADV	NTP binding site
V	xxTVHx	VHTVHE	VTVHEAQGATY	Nucleic acid binding
Va	FQGxE	VQGET	AQGATY	Coordination between NTP and nucleic acid binding site
Vb	VRxxDxx	IIARDSP	IQSSAAH	Nucleic acid binding
VI	VAxTRARx	VSLSRHTK	VALTRHTE	NTP binding site

The first column represents the conserved motif name present in all SF1 helicases. The second column represents the conserved amino acid residues in respective motifs (x is any amino acid). In contrast, the third column represents the motifs in TMV helicase (highlighted in yellow). The fourth column represents the motif sequence present in HEV helicase, the conserved residues are highlighted in yellow, and the fifth column represents the function of conserved motifs.

### Virtual screening of FDA-approved compounds

ATP, AGS, GTP, and the 136 FDA-approved compounds library were docked with a refined and minimised HEV-helicase structure. Five compounds were shortlisted from the virtually screened library (Table 1) based on their docking score, hydrogen bonds formed, and their interacting amino acid residues.

**Figure 2.**
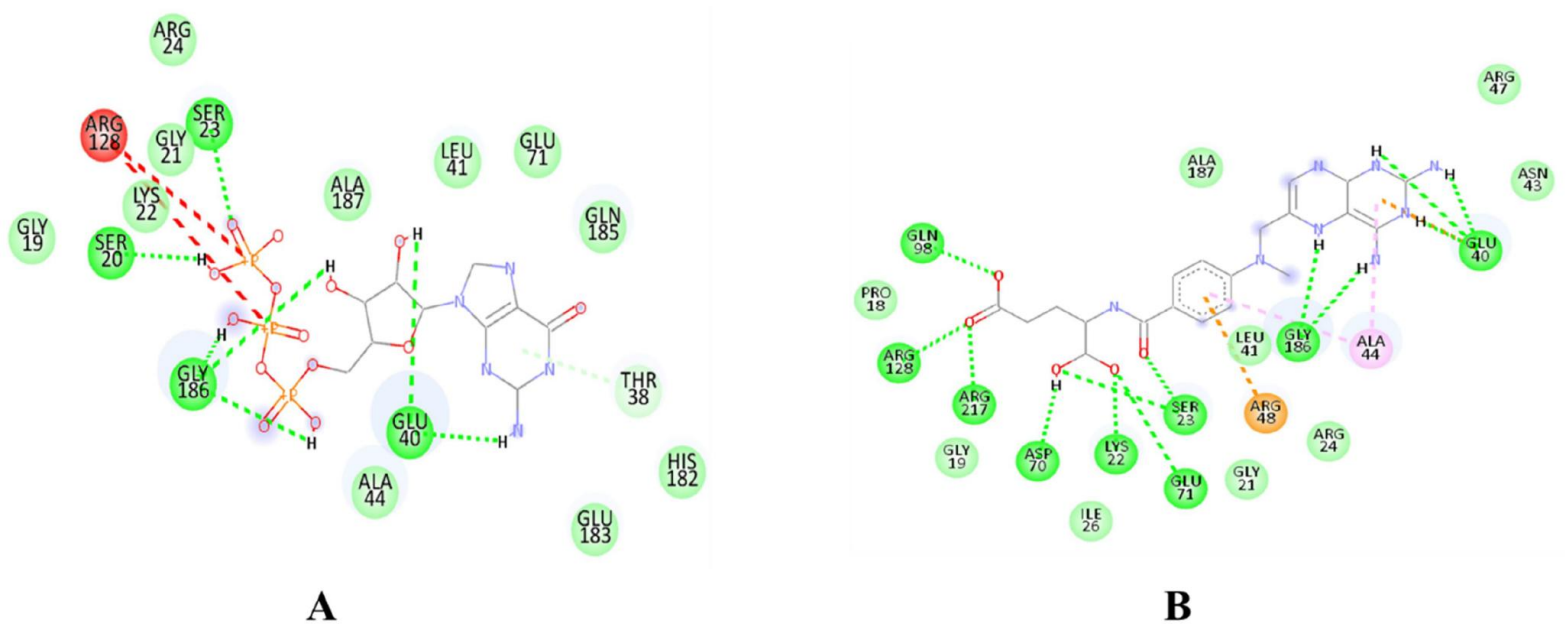
Protein–ligand interaction diagram- Figure- represents the interaction diagram of (A) GTP and (B) methotrexate with key amino acid residues at the binding pocket of HEV helicase. (A) Depicts hydrogen bonding and hydrophobic interactions between Helicase and GTP. (B) Depicts hydrogen bonding and hydrophobic interactions between Helicase and Methotrexate. The hydrogen bonds are represented with green dashed lines, and the interacting residues of HEV helicase are labelled.

The docking results showed that ATP, GTP, AGS, and the selected compounds form hydrogen bonds with conserved motifs of SF-1 helicases; also, the interacting residues, such as Pro18, Gly19, Ser20, Gly21, Lys22, Ser23, Arg24, Arg128, and Arg217 were predicted as binding site residues by COACH web server. Methotrexate formed one hydrogen bond with Gln98, Arg128, Arg217, Asp70, and Lys22, while Ser23, Gly186, and Glu40 formed two hydrogen bonds with methotrexate. The binding affinity of the selected compounds is given in Table 2, while the 2D protein–ligand interaction diagram of the selected compounds is shown in Figure 2. 2D Interaction diagrams of other ligands are provided in Supplementary materials (Figure S3).

**Table 2.** The table represents the ZINC ID, common name, and binding affinity of the ligands selected after the virtual screening of the FDA-approved library from the ZINC database. Rocaglamide and silvestrol were used as docking control.

ZINC ID	Ligand	Binding affinity
ZINC000001529323	Methotrexate	−8.6
ZINC000001530788	Cromolyn	−8.6
ZINC000003917708	Daunorubicin	−8.5
ZINC000002005305	5-Methyltetrahydrofolate	−8.3
ZINC000003833821	Prdl	−7.6
ZINC000005664046	Rocaglamide	−7.3
ZINC000095099283	Silvestrol	−7.3

### MD simulations

**Figure 3.**
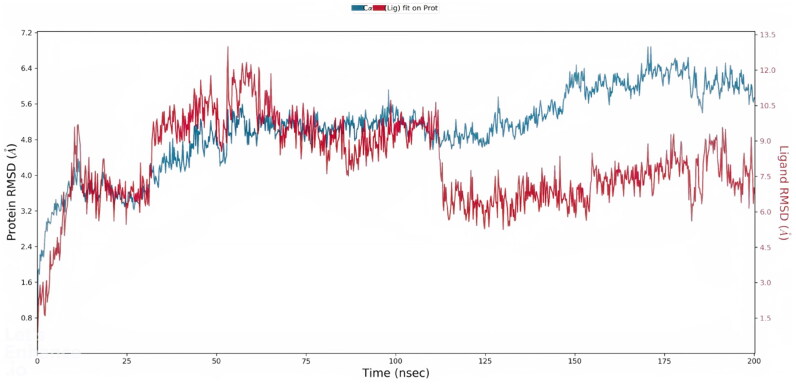
MD simulations analysis trajectory plot of HEV helicase-methotrexate bound complex. Figure 3 represents the RMSD of the helicase-methotrexate complex during 200 ns MD simulation.

The MD simulations for HEV helicase apo form complexed with methotrexate and levomefolic acid were computed for 200 ns. The helicase system containing an apo form had 34,089 atoms and 10,089 water molecules with zero net charges. Also, the final simulation box comprised 32 Cl ions, 57.69 (µM), and 28 Na ions, 50.460 (µM). The helicase-methotrexate system was composed of 30671 atoms and 8932 water molecules. Also, the system has a net zero charge, while the helicase-levomefolic acid system was composed of 27,348 atoms and 7824 water molecules. RMSD and RMSF values were calculated for both apo and complex structures. Generally, a lower RMSD value of the protein-ligand complex signifies a stronger binding of the ligand with the protein^38^. The apo-Helicase RMSD stabilised at 50 ns, the helicase-methotrexate’s RMSD value was stable after 100 ns, and the ligand RMSD was much lower than the protein RMSD, as shown below in Figure 3. A lower ligand RMSD signifies the stability of the protein-ligand complex throughout the simulation. The RMSD of helicase-levomefolic acid converged at around 200 ns. The RMSF values represent the local changes along the protein chain; the RMSF values of the apo-helicase fluctuated at about 3.6 Å, while RMSF values for helicase-methotrexate and helicase-levomefolic acid fluctuated at around 6.4 and 8 Å, respectively.

**Figure 4.**
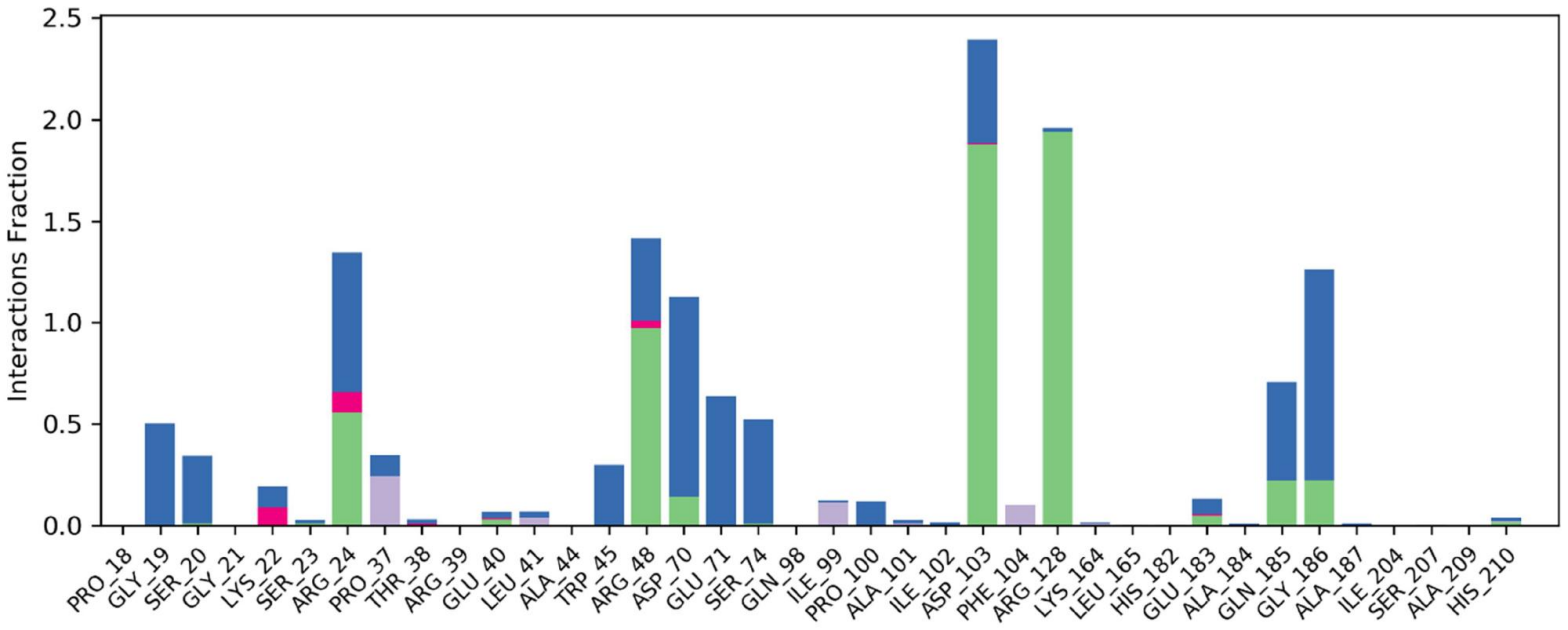
Protein–ligand contacts. The above figure represents protein-ligand contacts of the helicase-methotrexate complex during 200 ns MD simulation. The hydrogen bonds between helicase and methotrexate have been depicted in green, and water–water bridges have been depicted in blue colour; very few ionic interactions were observed between helicase and methotrexate, as illustrated in the figure, and the ionic interactions are displayed in red colour. Hydrophobic interactions are depicted in lavender colour.

**Figure 5.**
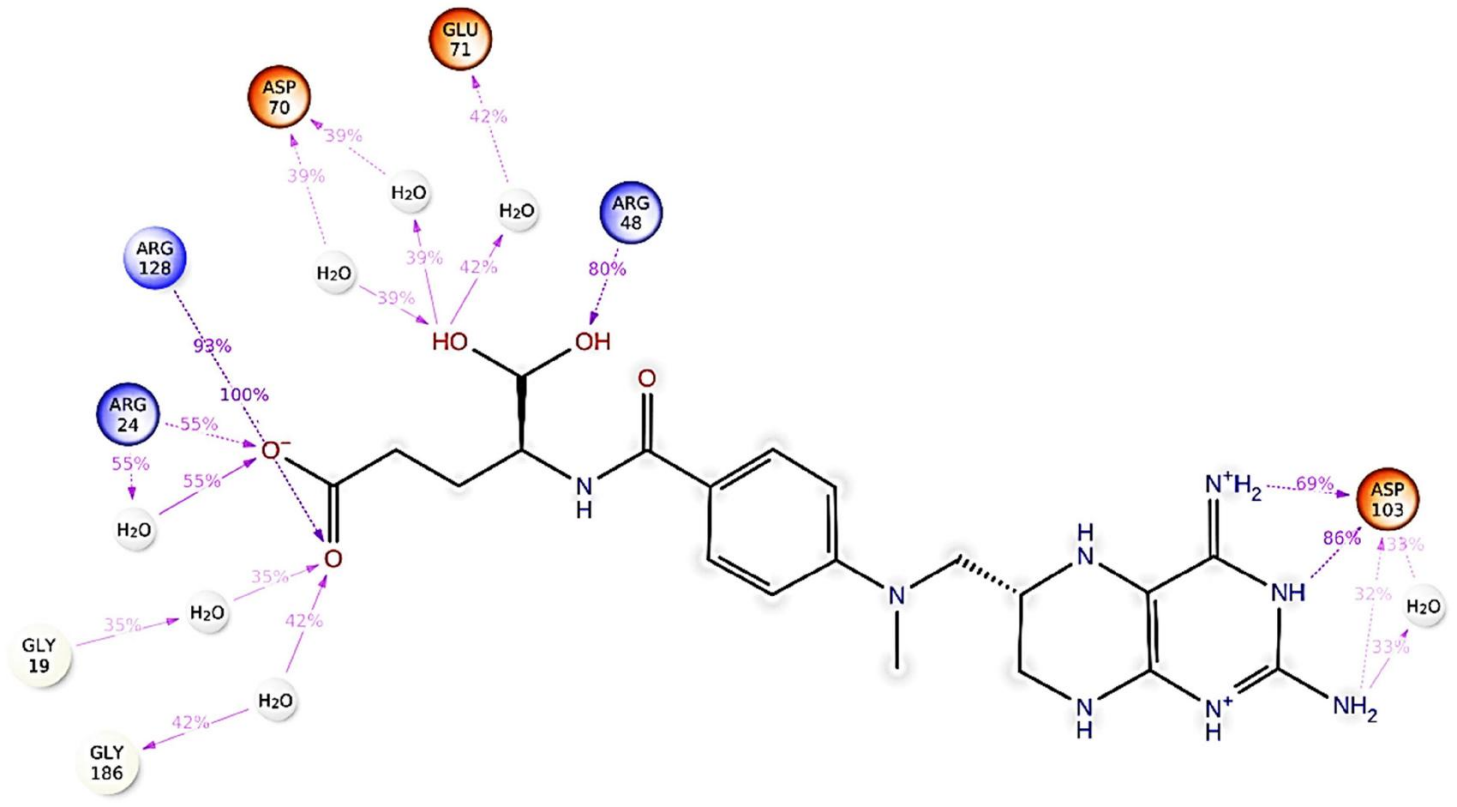
2D depiction of protein-ligand contacts. The figure above represents a 2D depiction of the percentage of protein-ligand contacts between helicase and methotrexate complex. Arg24, Arg128, and Arg48 showed the maximum interaction rate during the 200 ns MD simulation. Asp70 and Asp103 also showed significant interaction with the methotrexate.

Intermolecular hydrogen bonding and polar interactions are essential in analysing the binding affinity. The hydrogen bond formed during the 200 ns simulations was calculated for helicase-methotrexate and helicase-levomefolic acid. In helicase-methotrexate and helicase-levomefolic acid, the maximum number of hydrogen bonds formed were 8 and 7, respectively. Arg128, Arg24, Arg48, Asp70, Glu 71, Asp103, Gly19, and Gly186 form hydrogen bonds throughout the simulation, as depicted in Figure 4. Arg128 interacted 100% during the 200 ns MD simulation in the helicase-methotrexate complex, as illustrated in Figure 5. In the case of helicase-levomefolic acid, Lys22, Glu71, Asn97, His182, Gln185, and Asp199 formed hydrogen bonds during 200 ns simulation (Supplementary materials; Figure S4), but Asp199 was the major contributor to hydrogen bonding with levomefolic acid (199%) throughout the simulation (Supplementary materials; Figure S5).

### Cloning, expression, and purification of HEV helicase

**Figure 6.**
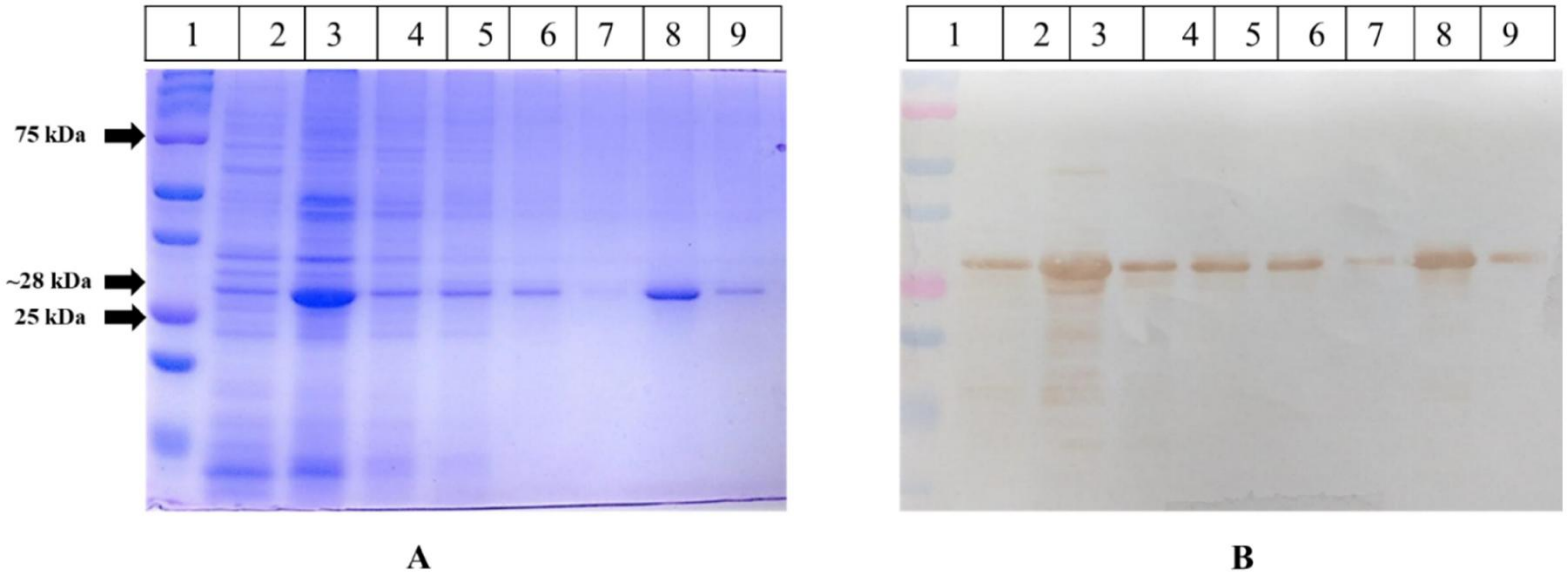
Ni-NTA (IMAC) protein purification of HEV helicase. Figure 6(A) represents Coomassie Brilliant Blue stained 12% SDS-PAGE. Lane 1 contains the pre-stained protein marker; Lanes 2 and 3 have the 0.5% NLS solubilised protein fraction sample; lane 4 contains flow-through; lanes 5, 6, and 7 include the wash. Lanes 8 and 9 have different elution fractions. Figure 6(B) represents the western blot analysis of the same samples. Lane 1 contains the pre-stained protein marker; Lanes 2 and 3 have the 0.5% NLS solubilised protein fraction sample; lane 4 contains flow-through; lanes 5, 6, and 7 include the wash. Lanes 8 and 9 have different elution fractions probed with anti-helicase-specific antibody in 1:3000 dilutions.

The protein was expressed and purified to study the enzyme kinetics and enzyme inhibition of the HEV helicase. The helicase gene of approximately 735 bp was cloned in the pET28a vector and confirmed by double digestion and DNA sequencing (data not shown). The recombinant HEV helicase-pET28a plasmid was transformed into BL21 cells for protein expression using SDS PAGE and confirmed by Western blotting (Figure 6). The protein was solubilised using different detergents, of which 0.5% NLS showed maximum protein solubilisation from inclusion bodies. The recombinant 27 kDa His-tagged HEV helicase protein was purified using IMAC Ni-NTA chromatography, fractionated on SDS PAGE (Figure 6(A); Lane 8), and validated by Western blotting using a helicase epitope-specific antibody (Figure 6(B); Lane 8).

### Enzyme kinetics

**Figure 7.**
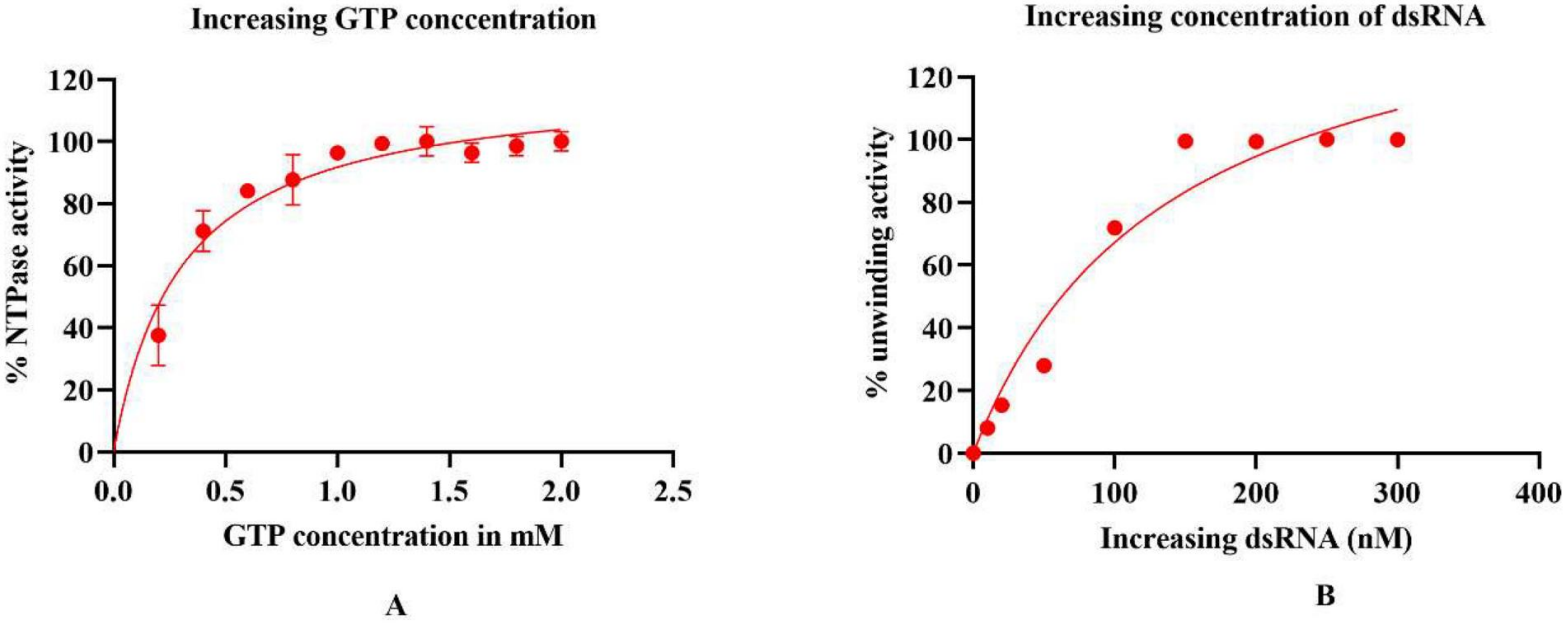
Effect of Substrate concentration on enzyme activity: (A) Michaelis Menten constant (K_m_) was calculated from the NTPase activity of the purified HEV helicase by varying the substrate (GTP) concentrations from 0 to 2.5 mM. The graph for NTPase activity was fitted using the Mechalis-Mentenn equation, and the calculated K_m_ value was found to be 0.35 mM (Figure 7(A)). The unwinding activity of purified HEV helicase was also determined using double-strand RNA as a substrate. Different concentrations of dsRNA ranging from 0 to 300 nM were used to calculate the K_m_ value. The graph was again fitted with the Mechalis–Menten equation, and the estimated K_m_ value for dsRNA as a substrate was found to be ∼100 nM. All data points in the graph represent the mean or average value of the triplicate readings, and the error bars indicate the standard deviation.

**Figure 8.**
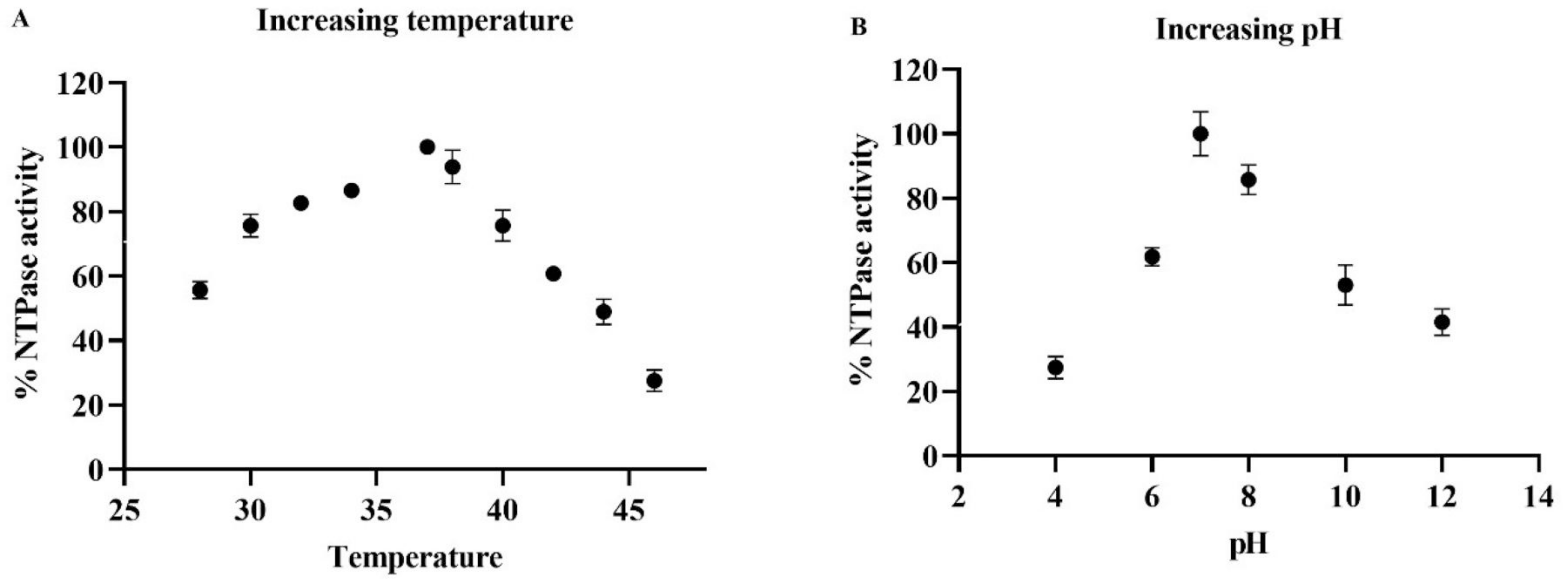
Effect of temperature and pH on enzyme activity: The above graph represents the effect of different temperatures and pH on NTPase activity.The protein and substrate concentrations were kept constant, and the reaction was incubated for temperatures ranging from 25 to 45 °C (Figure 8(A)), Also at a constant protein and substrate concentrations and varying pH from 2 to 14 (Figure 8(B)). The enzyme activity was measured as % NTPase. All the experiments were performed in triplicates. The data points in the graph represent the mean or average values, and the error bars indicate the standard deviation.

The enzyme kinetics of the purified helicase were studied using an NTPase assay and the unwinding assay. Various parameters were studied, including K_m_, temperature, and pH. The effect on enzyme activity was studied using two substrates, GTP and the double-stranded RNA. The GTP concentration was increased from 0 to 2.5 mM, and the % NTPase activity was determined. The K_m_ of the substrate or GTP was calculated using the Michelis-Menten equation, which is ∼0.35 mM (Figure 7(A)). Similarly, the concentration of another substrate, dsRNA, was increased from 0 to 400 nM, and the % unwinding ds RNA was calculated. The K_m_ was found to be ∼100 nM, and the calculated V_max_ was 150 nM (Figure 7(B)). The unwinding activity assay was conducted to estimate the optimum temperature by varying the temperature from 25 °C to 45 °C. The maximal activity was observed at 37 °C, above which the activity declined to indicate the enzyme’s degradation at higher temperatures (Figure 8(A)). Further, to determine the optimal pH of the reaction buffer, the enzyme assays were performed by varying pH from 4 to 12. A bell-shaped curve was observed when the pH was plotted against the activity, and the maximal activity was found at pH 7 (Figure 8(B)).

Post-optimisation, the NTPase assays for helicase were performed using 100 pmol of the enzyme, 0.4 mM GTP substrate, pH 7.0 at RT, and unwinding activity assay was conducted with 150 nM of dsRNA, 10 mM ATP, and 0.9 µM of helicase protein at RT. All the experiments were performed in triplicates, and the graphs were plotted using GraphPad Prism software (Boston, MA).

### Inhibition of HEV helicase enzyme activity

**Figure 9.**
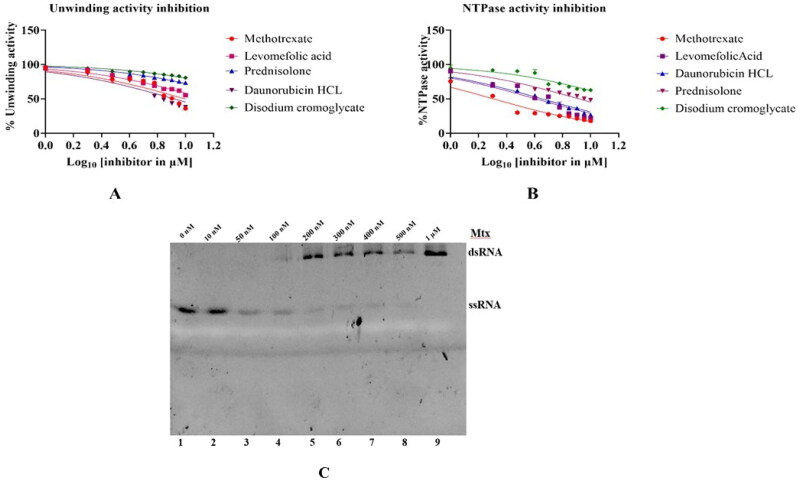
Cell-free NTPase and unwinding inhibition assay and IC_50_ determination of FDA-approved compounds. The graph represents the enzyme inhibition activity of the compounds evaluated using NTPase inhibition assay and unwinding activity inhibition assay. Figure 9(A) illustrates unwinding activity inhibition by FDA-approved compounds, and Figure 9(B) represents the inhibition profile by NTPase activity assay. The no compound control was taken as 100% HEV helicase activity, and no enzyme control was taken as 0% activity. All the experiments have been performed in triplicates. The statistical analysis was performed using a student t-test, and a *p* value <0.005 was considered statistically significant. *p < 0.0001; n.s *p* > 0.005. All experiments were performed in triplicates, and the graph was plotted in GraphPad Prism software. Figure 9(C) represents the effect of increasing methotrexate concentration on the unwinding activity. With the increase in methotrexate concentration, the unwinding activity is decreased, as represented by the increase in intensity of the dsRNA band. The samples were run on 10% Native PAGE, and the bands were visualised on a fluorescence (FAM) based gel dock system.

**Figure 10.**
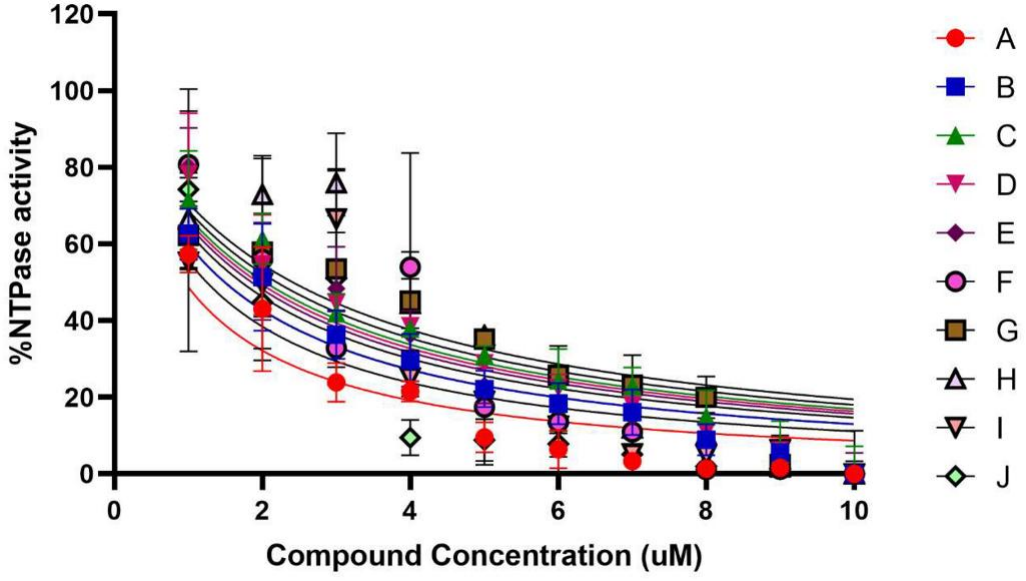
Cell-free NTPase and IC_50_ determination of ten novel compounds^41^. The graph represents the enzyme inhibition activity of the compounds evaluated using an NTPase activity inhibition assay. All the experiments have been performed in triplicates. The compounds were assessed at a concentration of 1–10 µM. The no compound control was taken as 100% HEV helicase activity, and no enzyme control was taken as 0% activity of HEV helicase. The graph’s data points represent the mean or average value of three readings, and the error bars indicate the standard deviation.

To identify the inhibitors against HEV helicase, five compounds, namely, Methotrexate, Levomefolic acid, Daunorubicin HCL, Prednisolone, and Disodium cromoglycate, were shortlisted after the virtual screening and evaluated for their inhibitory property by NTPase activity (Figure 9(A)) and the double-stranded RNA unwinding activity^32^ (Figure 9(B)). The inhibition kinetics for all the compounds was performed by varying the concentration of the inhibitors from 0 to 10 µM and estimating their effect on the activity of the helicase. The inhibition profile of the compounds using both assays was similar (Figure 9(A,B)), and the values are given in Table 3. The IC_50_ value of all the inhibitors was plotted using GraphPad Prism software (Boston, MA). Methotrexate, prednisolone, and levomefolic acid showed maximum inhibition from FDA-approved compounds. Along similar lines, 10 other putative compounds from previous literature^39^, were identified using *in silico* screening and assessed for their inhibitory property by NTPase activity assay (Figure 10). Further, the IC_50_ value of all the compounds using inhibition assay is given in Supplementary data Table S2. Methotrexate inhibited the HEV helicase NTPase and unwinding activity very efficiently in cell-free assays, as seen from IC_50_ values. Also, The unwinding activity inhibition was confirmed by running the samples on 10% Native PAGE, which shows that when helicase concentration is increased at a constant dsRNA concentration, the unwinding of the dsRNA occurs, resulting in the one smaller ssRNA band (Figure 9(C)). In contrast, on increasing methotrexate concentration, the helicase cannot unwind or break the dsRNA into ssRNA (Figure 9(D)). Levomefolic acid was found to be the second-best inhibitor in the cell-free assays, compound A (PubChem ID-BTB07890), compound B (PubChem ID-JFD02650) from previously published *in silico* screening also showed significant inhibition of NTPase activity in cell-free assays.

**Table 3.** IC50 determination from NTPase and unwinding inhibition assay.

IC_50_ values of different compounds using NTPase and unwinding assay		
Compound name	IC50 (µM) NTPase activity	IC50 (µM) Unwinding activity
Methotrexate	1.81	9.849
Levomefolic acid	3.65	13.92
Prednisolone	4.202	25.83
Daunorubicin HCL	7.663	8.253
Disodium cromoglycate	14.84	39.76

IC50 values of different FDA-approved compounds from NTPase inhibition assay and unwinding assay.

### Microscale thermophoresis analysis

**Figure 11.**
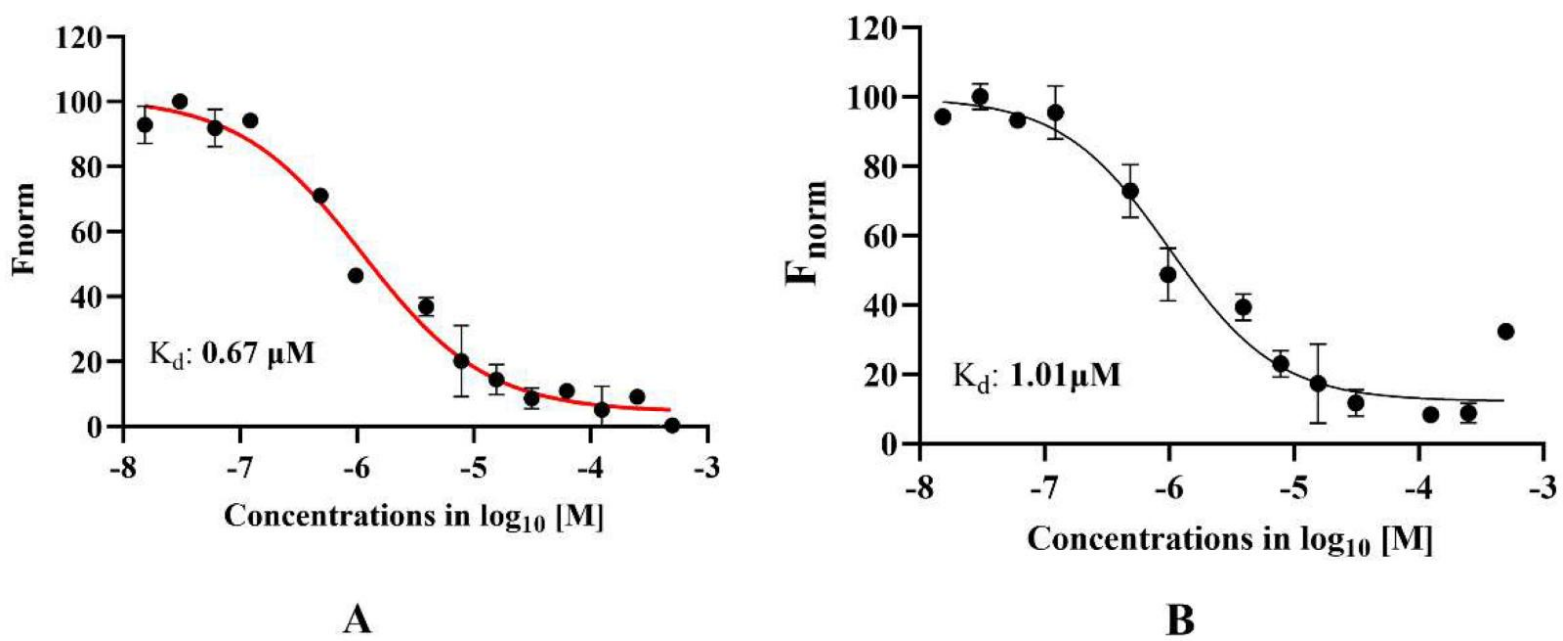
Microscale thermophoresis assay. The figure represents the dose-response curve for the binding interaction between Red-Tris NTA-labelled Helicase and Methotrexate (Figure 11(A)) and compound A (Figure 11(B)). The concentration of RED-Tris-NTA-labelled helicase was kept constant, and the concentration of methotrexate and compound A varied between 500 µM and 150 nM. The Y-axis represents the ΔF_norm_ fluorescence, and the X-axis represents the concentration of methotrexate and compound A, respectively. Here, the values represent the mean values of three independent experiments, and the error bar indicates the standard deviation.

After IC_50_ determination, the binding affinity of the selected compounds with purified HEV-helicase was determined using a microscale thermophoresis experiment. Of all compounds assessed on MST, Methotrexate showed the lowest binding constant (K_d_) value of 670 nM, while compound A showed a K_d_ value of 1.01 µM. The results obtained from the MST experiment agreed with the enzyme inhibition assays. At the same time, the compounds that did not show any interaction with the purified helicase were not considered for further studies. The dose-response curve of methotrexate and compound A is shown in Figure 11(A,B), respectively. The experiment was performed on the Monolith NT.11.5 instrument. All the MST experiments were performed in triplicates, and the significance of the data was checked using an unpaired t-test, and a *p* values <0.05 was considered statistically significant.

### Real-time quantitative PCR (qRT-PCR)

**Figure 12.**
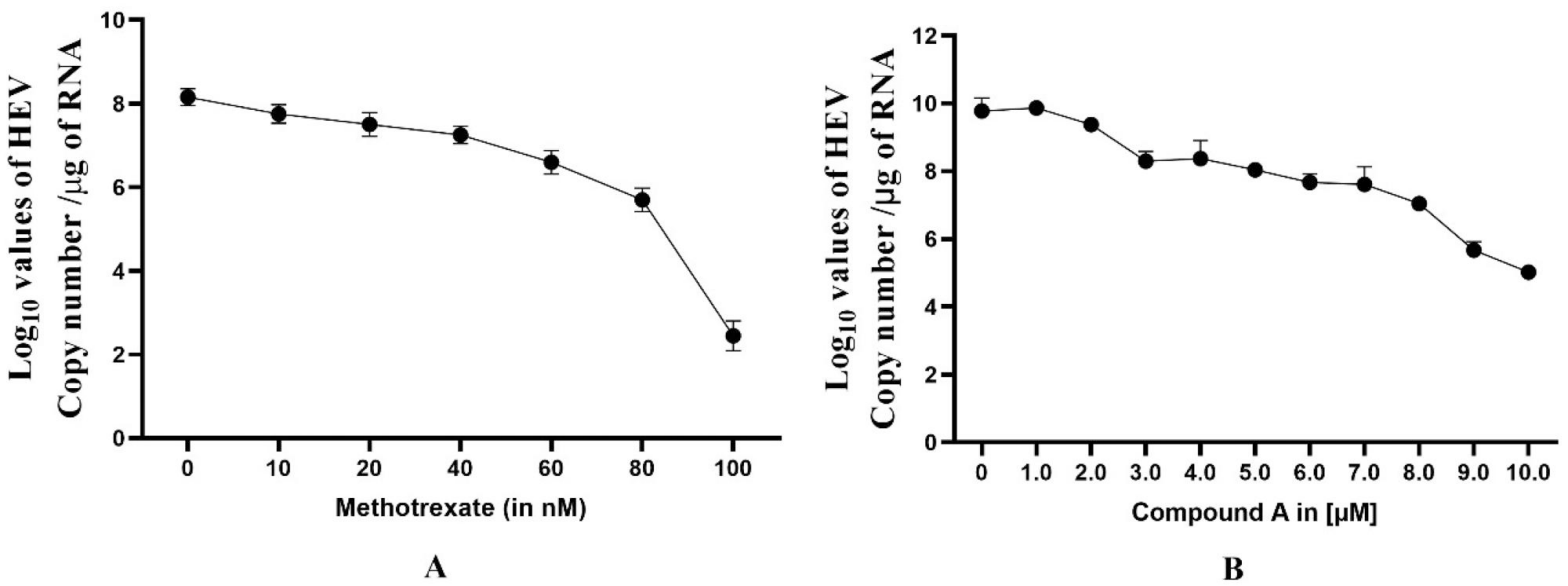
HEV Replicon Assay. Figure 12(A,B) represents the dose-dependent graphs that indicate the % inhibition of viral replication in HEV replicon-based assay in the presence of methotrexate and compound A. It was generated by quantifying luminescence, and each bar in the graph represents the mean value of triplicate, and the error bar indicates the standard deviation.

Out of all the compounds mentioned, methotrexate and compound A were selected for further studies due to their high affinity and low IC_50_ value compared to other compounds. The effect of methotrexate and compound A was further examined on HEV RNA replication using a transient culture of HEV. The cytotoxicity of methotrexate and compound A was checked using an MTT assay, and it was found to be < 100 nM for methotrexate and <25 µM for compound A. The Huh7 cells were transfected with HEV RNA and treated with different concentrations of methotrexate ranging from 0 to 100 nM, also 5 µM, and 10 µM of compound A. The RNA was extracted from the transfected cells, and the HEV RNA quantification was performed using HEV-ORF2-specific primers. Through qPCR, the viral RNA copies decreased from ∼4.2 × 10^8^ in untreated or mock samples to ∼4.2 × 10^1.6^ in the presence of 100 nM of methotrexate (Figure 12(A)) and ∼4.2 × 10^7.5^ to ∼ 4.2 × 10^3.5^ (Figure 12(B)). The reduction in RNA copies was attributed to the inhibitory property of the methotrexate that functioned as an inhibitor of the helicase. The graph represents the log_10_ RNA copy number per μg of total cellular RNA on the Y-axis and the concentration of methotrexate on the X-axis. The viral RNA copies were normalised with the host GAPDH level to equalise the amount of RNA.

### HEV replicon assay

**Figure 13.**
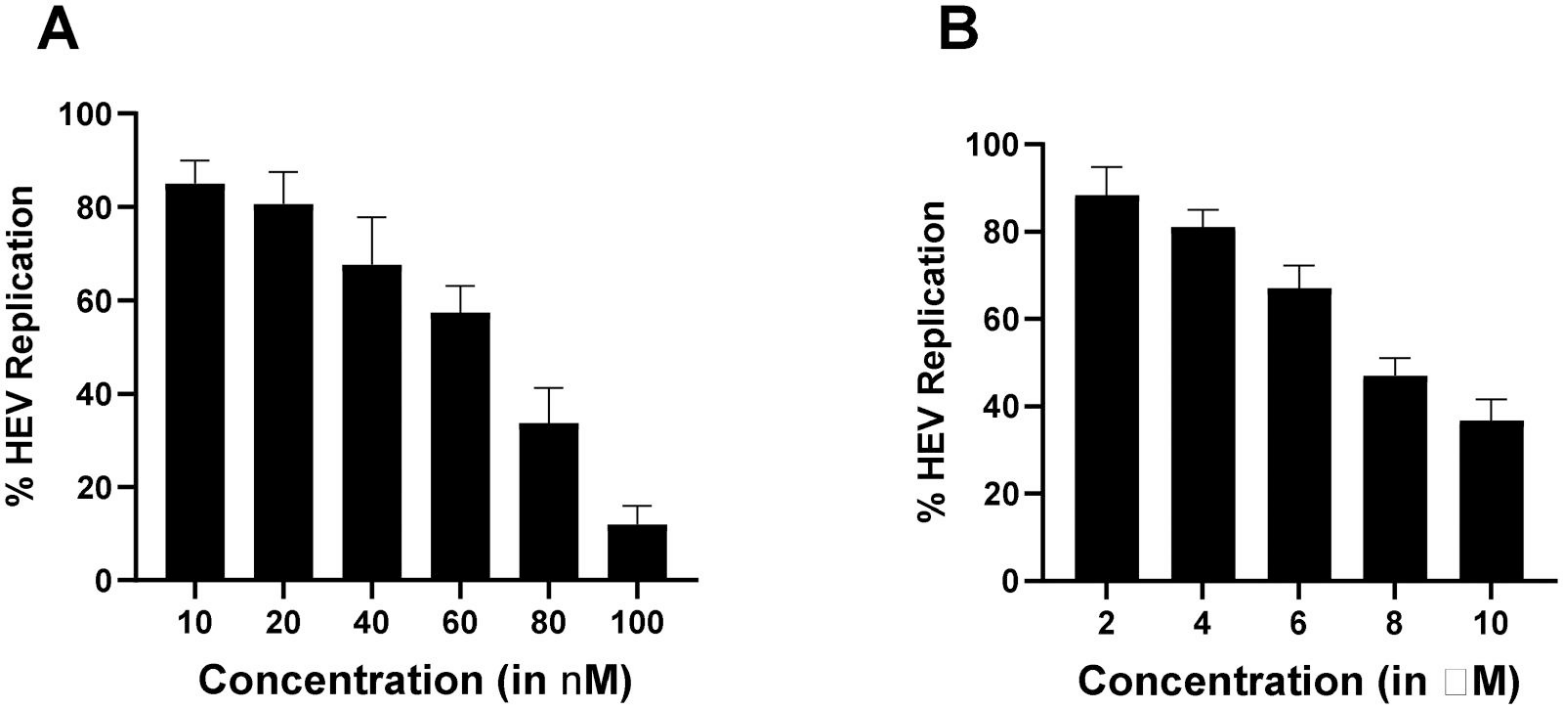
Effect of Methotrexate and compound A on HEV RNA Replication. The graph represents the reduction of viral RNA copy number upon varying the concentration of methotrexate (Figure 13(A)) and compound A (Figure 13(B)). Here, the values represent the mean values of three independent experiments, and the error bar indicates the standard deviation.

Methotrexate and compound A were selected as potential helicase inhibitors, and their effect was further studied on p6 HEV-Luc Replicon. The huh-7 cells were treated with methotrexate and compound A from 0 to 100 nM and 0 to 10 μM, respectively, and incubated for 72 h. It was observed that the treated cells inhibited the viral-replication-dependent luciferase activity (Figure 13). Furthermore, the IC_50_ of Methotrexate (Figure 13(A)) and compound A (Figure 13(B)) were found to be 73.12 ± 12.7 nM and 9.07 ± 2.9 μM, respectively (Figure 13). The IC_50_ of the compounds was determined using GraphPad Prism version 10.0.0 (Boston, MA). The experiments were performed in triplicates; each bar in the graph represents the mean value, and the error bar indicates the standard deviation.

### Immunofluorescence assay (IFA)

**Figure 14.**
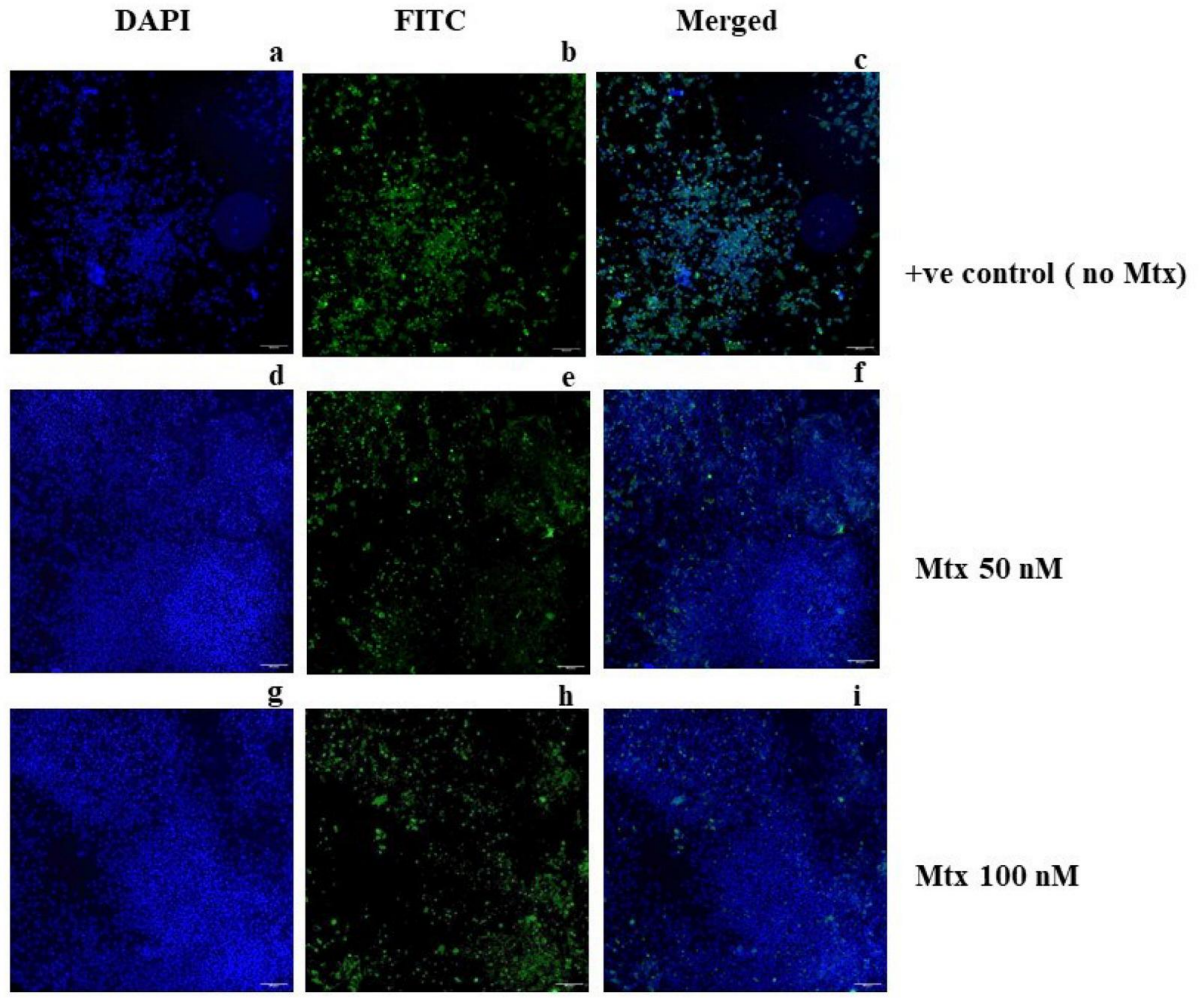
Effect of Methotrexate on HEV Replication using IFA. The cells were infected with the virus without an inhibitor as a positive control; panel (a) represents huh-7 cells stained with DAPI, (b) represents huh-7 cells stained with FITC, and (c) represents the merged image of DAPI and FITC. In another experiment, cells were similarly treated with the virus with an addition of 50 nM Mtx, where (d) represents huh-7 cells treated with DAPI, (e) represents FITC-treated cells, and (f) represents the merged image of DAPI and FITC. Further, in a similar experiment, the cells were treated with 100 nM of Mtx, and the panels (g), (h), and (i) represent DAPI, FITC, and merged images, respectively.

**Figure 15.**
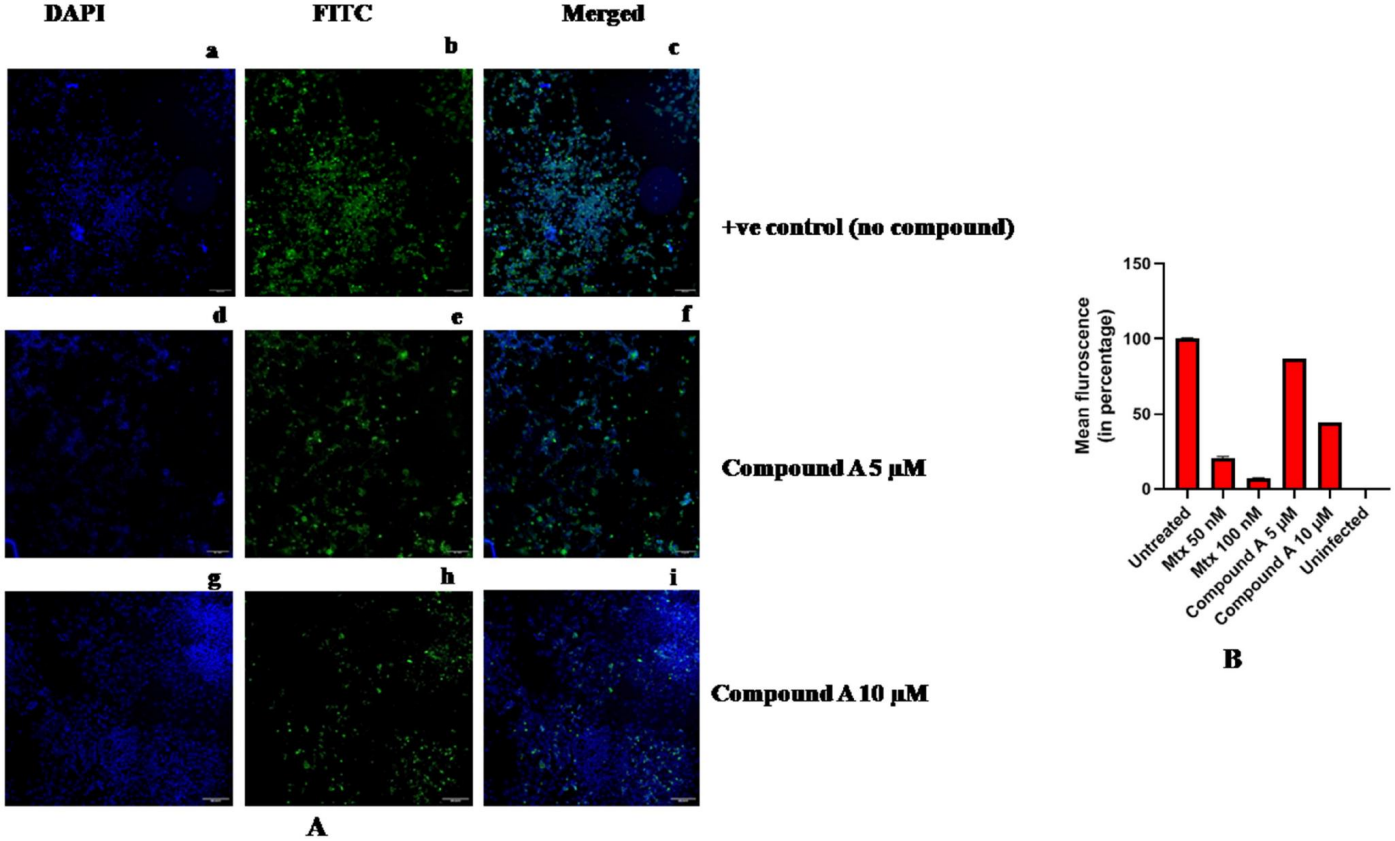
(A) Effect of Compound A on HEV Replication using IFA. The cells were infected with the virus without an inhibitor as a positive control, and panel (a) represents huh-7 cells stained with DAPI, (b) represents huh-7 cells stained with FITC, and (c) represents the merged image of DAPI and FITC. In another experiment, cells were similarly treated with the virus with 5 µM Compound A addition, where (d) represents huh-7 cells treated with DAPI, (e) represents FITC-treated cells, and (f) represents the merged image of DAPI and FITC, respectively. Further, in a similar experiment, the cells were treated with 10 µM of compound A, and the panels (g), (h), and (i) represent DAPI, FITC, and merged images, respectively. (B) Fluorescence quantification of infected and treated huh-7 cells. The graph above represents the fluorescence quantification data of the IFA experiment. The graph represents % HEV replication inhibition by the inhibitors tested. Untreated represents huh-7 cells infected with the virus without any drug treatment, while two concentrations of Mtx were tested (50 nM and 100 nM). At 50 nM Mtx concentration, ∼81% HEV replication inhibition was observed. At 100 nM Mtx concentration, ∼94% HEV replication inhibition was observed. Two concentrations of compound A, 5 µM and 10 µM were tested; at a concentration of 5 µM, compound A showed ∼14% HEV replication inhibition, while at a 10 µM concentration of compound A, ∼57% HEV replication inhibition was observed. The data points in the graph represent mean or average values from three different panels.

The effect of methotrexate and other compounds was also seen in untreated and treated cells through immunofluorescence using HEV-ORF2 epitope-specific antibody^26^^,^^36^. Compared to untreated cells, a decrease in ORF2 expression was observed in methotrexate-treated cells (Figure 14) and compound A-treated cells (Figure 15(A)). The total fluorescence was quantified using the multi-wavelength cell scoring module of the MetaXpress Software, and the fluorescence level was decreased from 100% to ∼6% in the 100 nM methotrexate-treated cells. It has been observed that there was ∼94% inhibition of HEV replication in the presence of 100 nM of methotrexate, while compound A showed ∼45% inhibition at 10uM (Figure 15(B)), which is relatively high to be considered as an inhibitor. The graph represents the mean value of total fluorescence from three different panels.

## Discussion

Although effective vaccines can prevent viral infections, their protective efficacy may be inconsistent due to specific mutations in viruses. Notably, the recently approved vaccines for SARS-CoV2^40^ have conferred short-lived immunity and shown diminishing protection against its highly pathogenic variants of concern (VOCs)^41^. Unfortunately, while China’s only licenced HEV vaccine is inaccessible to other countries, there is no approved anti-HEV drug except RBV. Thus, there is an urgent need to develop anti-HEV drugs to treat chronic hepatitis and to minimise the mortality rates in high-risk populations, like pregnant women and immune-compromised patients.

The HEV helicase has multienzyme functions, such as RNA duplex unwinding, NTPase, and RNA 5′-triphosphatase activities. The ability of helicase to bind and hydrolyse NTPs, dNTPs, and 5′-RNA-triphosphate suggests that its binding site is determined primarily by the triphosphorylated nucleotides. Helicase of Single-stranded RNA viruses’ have RNA 5′-triphosphatase activity associated 5′ capping of mRNA which is a well-known mechanism^42^^,^^43^. In contrast, because HEV is not known to have any other RNA 5′-triphosphatase domain, it seems reasonable to suggest that HEV employs its helicase to mediate the first step of 5′ cap synthesis through *γ*-phosphatase activity. In addition, the enzyme’s ability to unwind RNA duplexes with 5′ overhangs and having sequence-independent RNA 5′-triphosphatase activity suggests its role in forming a 5′ cap structure. We were also able to confirm the unwinding activity of Helicase using fluorescent and unwinding assays based on the difference in mobility of single and double-stranded RNA. Therefore, taken together, inhibition of helicase warrants abrogation of the methylation and unwinding of viral RNA duplex. Thus, the indispensable role of HEV helicase in its RNA replication and life cycle makes it an important drug target for the treatment of hepatitis E. Previously, several helicase-inhibitor drugs have been developed against Coronavirus^44–47^ Zika virus^48^, and dengue virus^49^. Helicase is also evidenced to be an essential target, as seen in the case of Herpes Simplex Virus (HSV)^50^^,^^51^, where the FDA has approved Amenamevir (ASP2151) and Pritelvir AC316^50,^^51^.

In this study, we first used the refined and validated 3D model of HEV helicase for structure-based virtual screening of the FDA-approved library of compounds, including those from ZINC, PubChem, and the Maybridge database. Following molecular docking and MD simulation analysis, the selected top five compounds were evaluated for inhibition of helicase enzyme activity, using *in vitro* assays and HEV cell culture model. Of these, methotrexate, the FDA-approved drug, was found to be the most potent HEV helicase inhibitor. In a recent *in silico* study, methotrexate has also been identified to bind to SARS CoV-2 helicase^44^^,^^52^^,^^53^. Therein, the effective concentrations of methotrexate used to inhibit the SARS-CoV-2 replication were the same as those used for human therapies^54^. In this study, we have shown replication inhibition of HEV RNA by Methotrexate at 100 nM, a comparatively much lower concentration for treating SARS CoV-2 infection. Methotrexate showed a strong binding affinity to purified HEV helicase and inhibited its enzymatic activity, validated by *in vitro* and virus-cell culture studies. Nonetheless, high-resolution crystal structures of helicase-bound complexes can further help identify new inhibitors of HEV. However, experimental validation of the inhibitory effect of methotrexate on the HEV life cycle in animal models of HEV would be the future scope.

## Conclusion

To identify an inhibitor against HEV, HEV RNA helicase was selected as a target. Virtual screening and MD simulations were performed to screen top-scoring drugs from the ZINC library of over 0.2 million compounds. In vitro NTPase and unwinding assay showed that methotrexate inhibited the HEV helicase NTPase and unwinding activity with an IC_50_ value of 1.81 and 9.84 µM, respectively. Binding analysis using MST also showed methotrexate interacts with purified helicase strongly with a K_d_ value of 670 nM. Besides screening FDA-approved drugs, we also evaluated the inhibitory potential of 10 previously published compounds using NTPase assay, from which compound A showed significant NTPase activity inhibition with an IC50 value of 0.94 µM and 1.01 µM in MST binding analysis. Methotrexate and compound A were further evaluated to inhibit the HEV transient culture system, and methotrexate showed a 90% reduction in viral RNA copies number. Compound A showed a ∼50% reduction in viral RNA copies number. Hence, it is concluded that methotrexate and compound A can be potent drug-like inhibitors against HEV replication, though they need further validation and *in vivo* studies.

## Supplementary Material

Supplemental Material
