# Supplementary material for: Methotrexate, an anti-inflammatory drug, inhibits Hepatitis E viral replication
Source: J Enzyme Inhib Med Chem. 2023 Nov 17;38(1):2280500. doi: 10.1080/14756366.2023.2280500 (PMC11003484; doi:10.1080/14756366.2023.2280500)
Supplement: Supplemental Material [file IENZ_A_2280500_SM4134.pdf]

Supplementary material

Identification of Hepatitis E virus helicase inhibitorspreventHEV- RNA replication.

Akash Kumar[1], Preeti Hooda[1], Anindita Puri[1], Radhika Khatter[1] Mohammed S. Al-Dosari[2], Neha Sinha[3], Mohammad K. Parvez[2]* and Deepak Sehgal[1]*

[1]Virology lab, Department of Life Sciences, Shiv Nadar Institution of Eminence, Gautam Buddha Nagar 201314, India

[2]Department of Pharmacognosy, College of Pharmacy, King Saud University, Riyadh 11451, Saudi Arabia.

[3]Department of Infectious Diseases and Microbiology, School of Public Health, University of Pittsburgh, Pittsburgh, PA 15261, USA.

*Corresponding author.

Keywords: Hepatitis E virus helicase RNA replication antiviral methotrexate

Rank	C-score	Cluster size	PDB hit	Ligand name	Consensus binding residues
**1**	0.53	26	3wrxC	AGS	18,19,20,21,22,23,24,98,127,128,186,188,217
**2**	0.33	17	5hfdB	ALE	18,19,22,71,98,128,186,217
**3**	0.08	8	2gjkA	Mg	23,71,186
**4**	0.01	1	5fhfA	ALE	22,23
**5**	0.01	1	2a5yC	MG	23,95
**5**	0.01	1	3GPLA	3GPLA00	102,103,104,169,180,182,183

Table 1. Prediction of ligand binding site. The table represents the prediction of the ligand binding site of HEV helicase. The ligand binding site prediction was done using the COACH web server. COACH is a meta server- approach to protein-ligand binding site prediction. The best binding site was ranked based on the C-score and cluster size. The residues of Rank-1 having largest cluster size contains some of the conserved motifs of SF1 helicase and are similar with the binding pocket/active site of the TMV helicase.

Compound	PubChem ID	IC50
A	BTB07890	0.9492
B	JFD02650	1.49
C	CD07424	2.038
D	SPB07861	1.949
E	HTS03126	1.856
F	RDR03130	1.719
G	HTS11136	2.194
H	JFD02375	2.411
I	KM07751	1.496
J	RJC03167	1.249

Table 2. IC50 determination from NTPase inhibition assay of previously published compounds (40). The table represents IC50 values of 10 (compound A to compound J) published previously. The Malachite green phosphatase assay was performed to study their inhibitory potential.

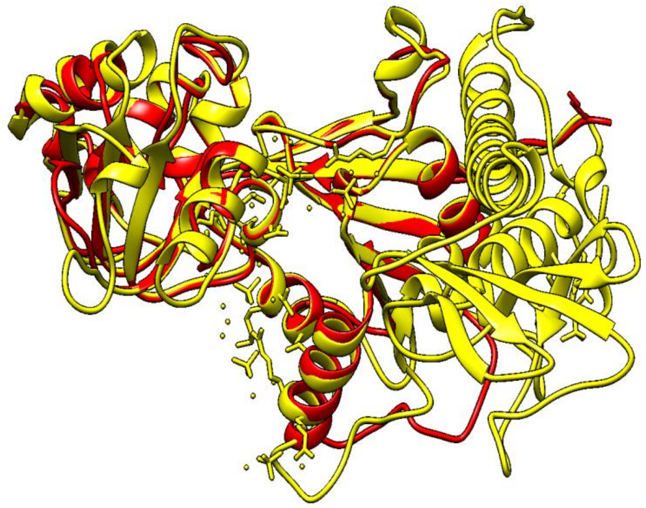

Figure-S1 Structure comparison of HEV helicase and TMV helicase. The RMSD value for structure comparison was 0.96 Angstrom. For structural superimposition UCSF chimera was used The PDB structure of TMV Helicase (PDB ID:3VKW) was used as a reference structure.

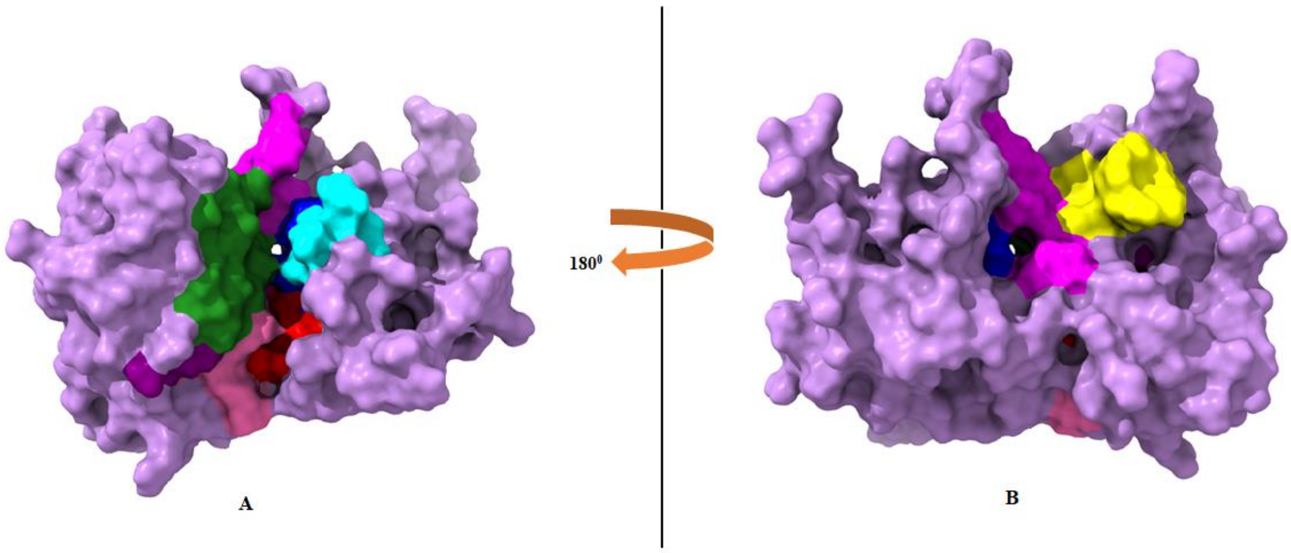

Figure-2 (A) Conserved SF1 helicase motifs present in HEV helicase represented in different colours. The binding cleft of HEV Helicase as predicted by COACH and METAPOCKET

web server is formed by Pro18, Gly19, Ser20, Gly21, Lys22, Ser23, Arg24, Gln98, His127, Arg128, Gly186, Thr188, Arg217. (B) 180° rotated image showing the NTP binding site of HEV helicase.

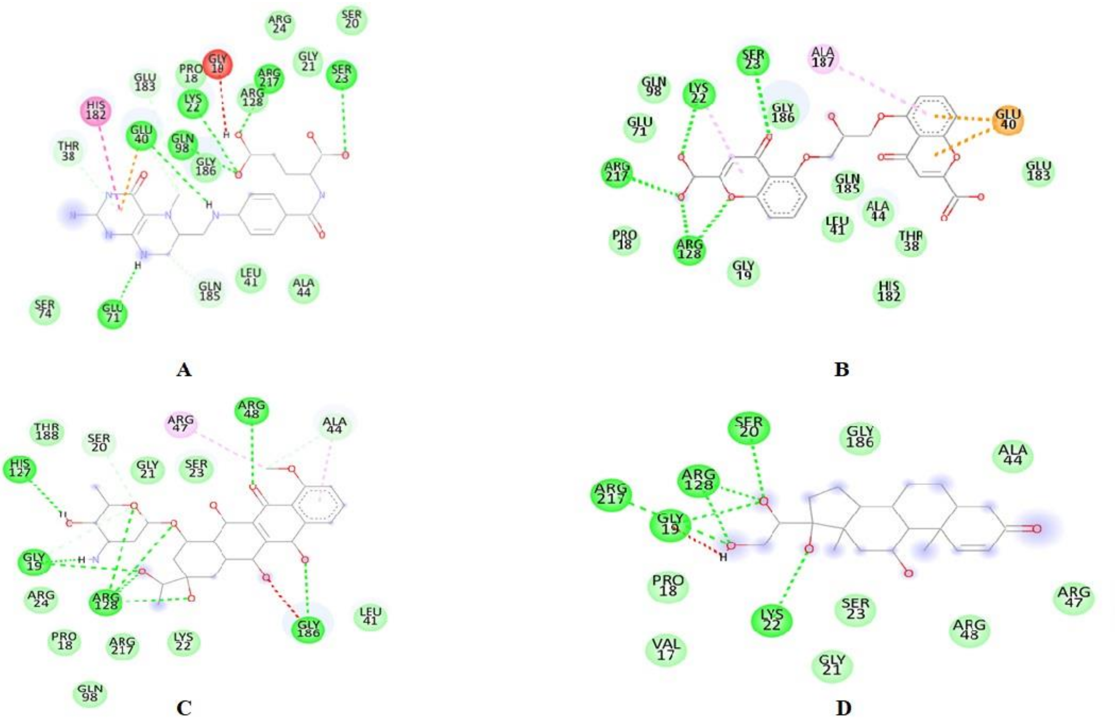

Figure-3 Protein-ligand interaction diagram- The figure represents the interaction diagram of (A) helicase-levomefolic acid and (B) helicase- prednisolone, (C) helicase daunorubicin HCL and (D) helicase-disodium cromoglycate with key amino acid residues at the binding pocket of HEV helicase. The hydrogen bonds are represented with green dashed lines, and the interacting residues of HEV helicase are labeled.

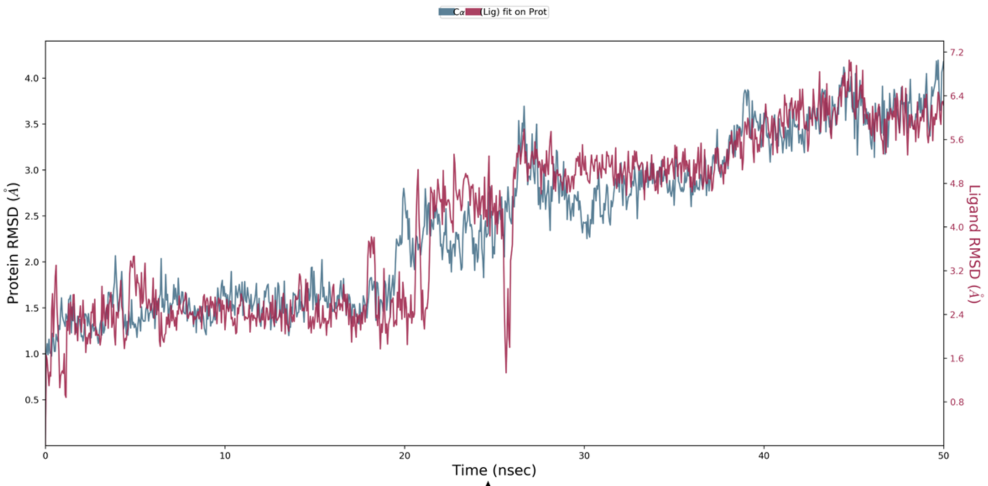

A

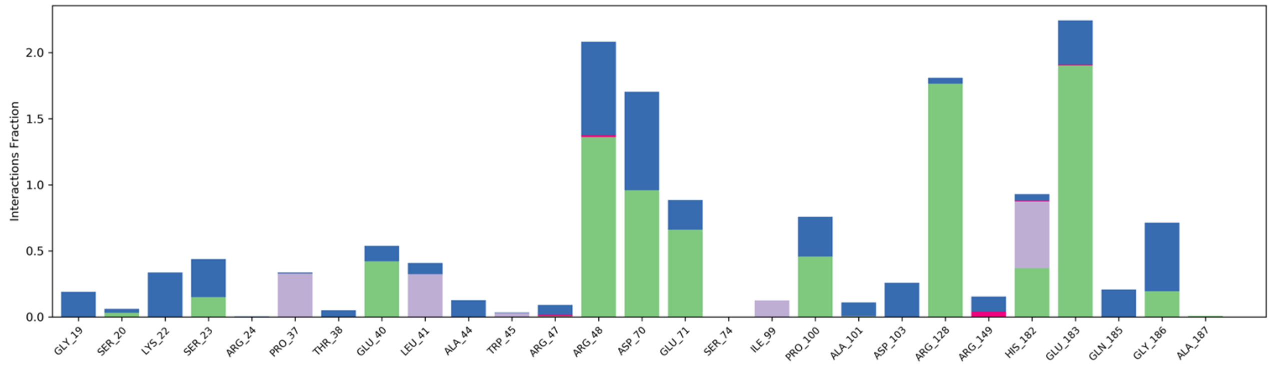

B

Figure-4 MD simulations analysis trajectory plot of HEV helicase-levomefolic acid bound complex. (A) Represents the RMSD of the helicase-levomefolic acid complex during 200 ns MD simulation, The RMSD values converges at around 200 ns. The figure (B) represents protein-ligand contacts during the 200 ns MD simulation in between helicase and levomefolic acid, 6 major hydrogen bonds were formed in between helicase and levomefolic acid were formed during the course of MD simulation as represented with green bars in the figure (B).

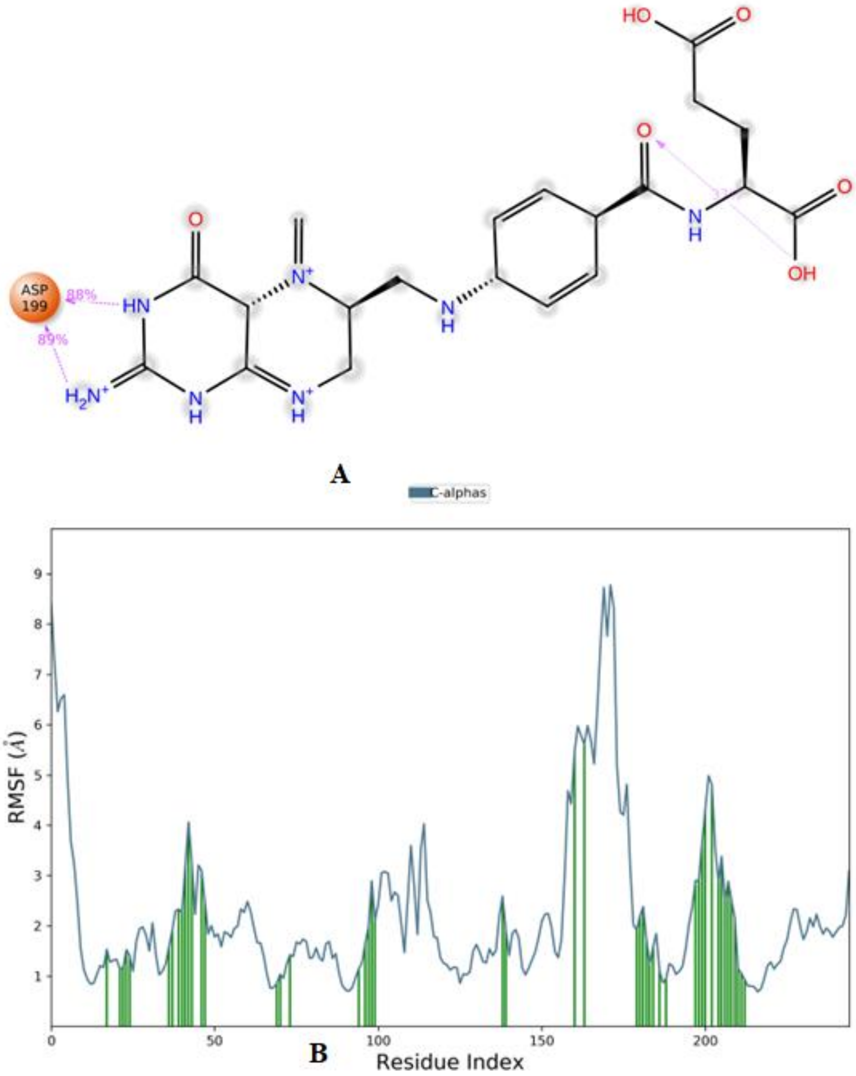

Figure-5 MD simulations analysis trajectory plot of HEV helicase-methotrexate bound complex. Figure (A) represents a 2-D depiction of the percentage of the protein-ligand contacts of the helicase-levomefolic acid bound complex during 200 ns simulation. Figure (B) Represents the RMSF values of the helicase-levomefolic acid complex during 200 ns MD simulation.
